# Somatic deletion of the *NF1 *gene in a neurofibromatosis type 1-associated malignant melanoma demonstrated by digital PCR

**DOI:** 10.1186/1476-4598-5-36

**Published:** 2006-09-10

**Authors:** Albert Rübben, Birke Bausch, Arjen Nikkels

**Affiliations:** 1 Department of Dermatology, University Hospital RWTH Aachen, Pauwelsstrasse 30, D-52074 Aachen, Germany; 2 Department of Nephrology, Albert-Ludwigs-University, Hugstetter Str. 55, D-79106 Freiburg, Germany; 3 Department of Dermatopathology, University Medical Center, Sart Tilman, B-4000 Liège, Belgium

## Abstract

**Background:**

Neurofibromatosis type 1 (NF1) is the most common hereditary neurocutaneous disorder and it is associated with an elevated risk for malignant tumors of tissues derived from neural crest cells. The *NF1 *gene is considered a tumor suppressor gene and inactivation of both copies can be found in NF1-associated benign and malignant tumors. Melanocytes also derive from neural crest cells but melanoma incidence is not markedly elevated in NF1. In this study we could analyze a typical superficial spreading melanoma of a 15-year-old boy with NF1 for loss of heterozygosity (LOH) within the *NF1 *gene. Neurofibromatosis in this patient was transmitted by the boy's farther who carried the mutation *NF1 *c. 5546 G/A.

**Results:**

Melanoma cells were isolated from formalin-fixed tissue by liquid coverslip laser microdissection. In order to obtain statistically significant LOH data, digital PCR was performed at the intragenic microsatellite IVS27AC28 with DNA of approx. 3500 melanoma cells. Digital PCR detected 23 paternal alleles and one maternal allele. Statistical analysis by SPRT confirmed significance of the maternal allele loss.

**Conclusion:**

To our knowledge, this is the first molecular evidence of inactivation of both copies of the *NF1 *gene in a typical superficial spreading melanoma of a patient with NF1. The classical double-hit inactivation of the *NF1 *gene suggests that the NF1 genetic background promoted melanoma genesis in this patient.

## Background

Neurofibromatosis type 1 (NF1; MIM# 162200) is an autosomal dominant neurocutaneous disorder characterized by multiple café-au-lait macules (CALMs) visible early in childhood and by development of neurofibromas in adult patients [1]. Besides, NF1 is associated with various malignant tumors such as malignant schwannoma (neurofibrosarcoma), medulloblastoma, astrocytoma and pheochromocytoma. The birth incidence of NF1 lies between 1/3000 and 1/3500 [1,2]. The disease is caused by mutations which inactivate one neurofibromin gene on the long arm of chromosome 17 (17q11.2) in the germline of affected patients. The protein neurofibromin encoded by the *NF1 *gene is a RAS-specific GTPase-activating protein that functions as a negative regulator of the RAS pathway [3]. It can be considered a tumor suppressor gene as inactivation of both copies of the *NF1 *gene can be found in NF1-associated malignant schwannoma and pheochromocytoma [4,5]. Inactivation of both *NF1 *alleles as well as loss of heterozygosity (LOH) of microsatellite DNA within the *NF1 *gene could also be demonstrated in benign NF1-associated neurofibroma [6-8]. NF1-associated neurofibroma, malignant schwannomas, medulloblastoma, astrocytoma, and pheochromocytoma derive from cells of neural crest origin. Although melanocytes derive from neural crest cells as well, melanoma incidence does not seem to be markedly elevated in NF1. In Europe, melanoma incidence lies around 10/100,000/year and melanoma has been found in 0.1–5.4% of NF1 patients [9-11]. Interestingly, it seems that melanomas tend to develop at younger age in NF1 patients which has been interpreted as indication of a non-fortuitous association [11]. Mutations or LOH at the *NF1 *gene are rare (≈5%) in typical malignant melanoma but could be demonstrated in 67% of desmoplastic neurotropic melanoma which represents a rare melanoma variant [12]. In NF1-associated melanoma, LOH has been reported only once in a melanoma displaying an atypical anal localization [13].

We hereby want to report the first molecular evidence of inactivation of both copies of the *NF1 *gene in a typical superficial spreading melanoma of a 15-year-old boy with NF1. Data were generated by combining liquid coverslip laser microdissection, microsatellite analysis and digital PCR [14-16]. This novel technical approach was necessary as LOH analysis by PCR of small formalin-fixed and paraffin-embedded tissue specimens is prone to generate false positive LOH results [17,18].

## Results

### Clinical features of the analyzed patient

Since early childhood the 15-year-old boy of Indonesian origin has developed multiple café-au-lait macules (CALMs) on his trunk and extremities. He further demonstrated freckling in the axillary. Likewise, the boy's father displayed multiple CALMs, freckling in the axillary, and several histologically proven neurofibromas of the skin as well as a spinal neurofibroma. Diagnosis of neurofibromatosis type 1 was established in both patients according to the NIH diagnostic criteria [19]. The *NF1 *mutation c. 5546 G/A was identified in the farther and in the patient (data not shown). This mutation which has already been described in several patients changes arginine to glutamine at codon position 1849 and induces skipping of exon 29 [20-22].

**Figure 1:**
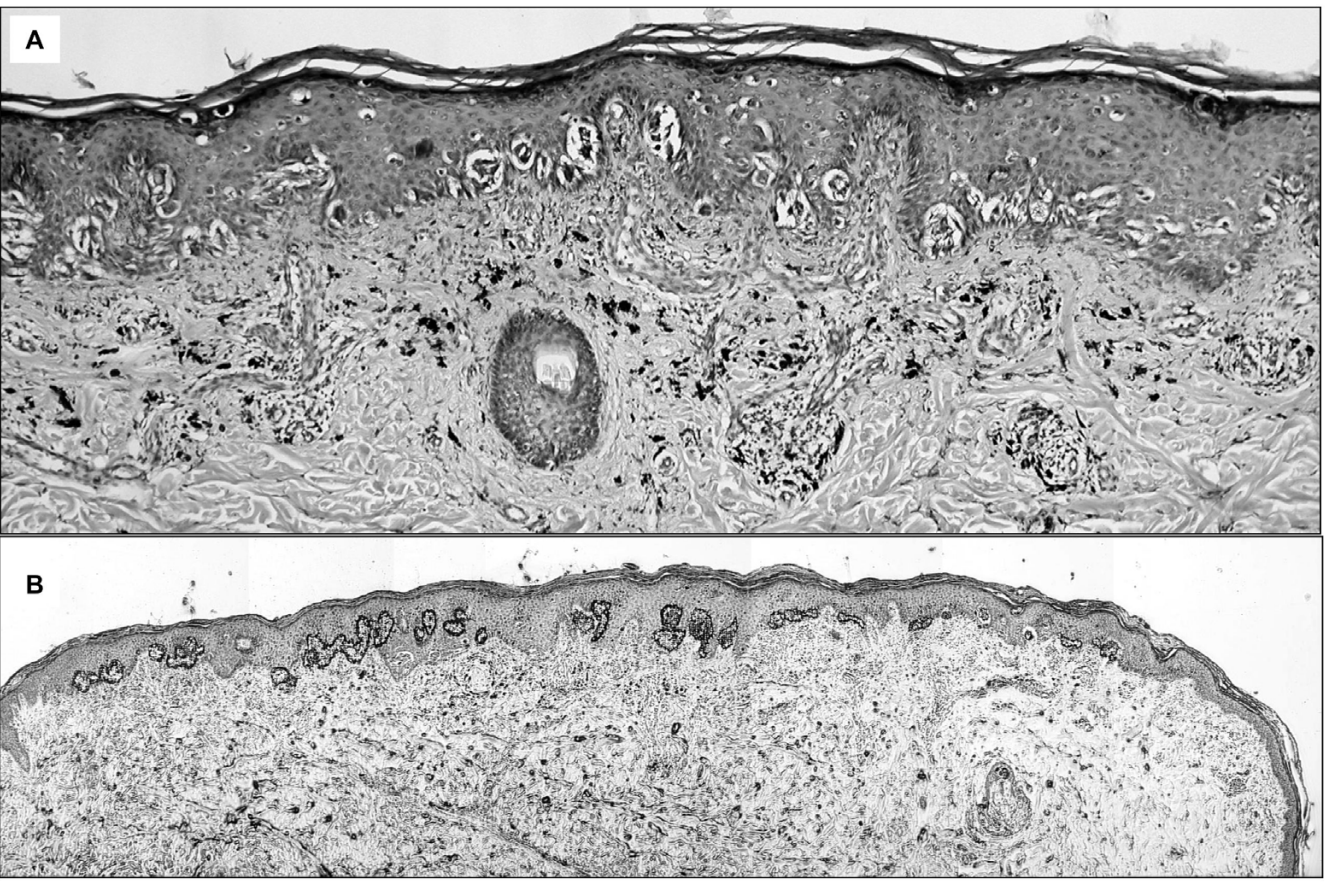
**Analyzed melanoma specimen**. A: H&E stained section of the analyzed superficial spreading melanoma (X10). B: Section after liquid coverslip laser microdissection with removal of melanoma cells (H&E, ×4).

The boy reported that he had had a pigmented mole on his left calf for several years but that he had observed growth and colour changes of the mole in the last six months before admission. The lesion was not associated with CALMs or with segmental pigmentation changes. Clinically, Spitz nevus or malignant melanoma was suspected. The lesion was removed and formalin-fixed for routine histopathological analysis. Histological examination revealed superficial spreading melanoma, Clark-Level II-III, tumor thickness 0.375 mm (pT1a, N0, M0), disease stage IA according to AJCC-UICC classification (Fig. 1A).

### Identification of paternal and maternal microsatellite alleles on 17q

**Figure 2:**
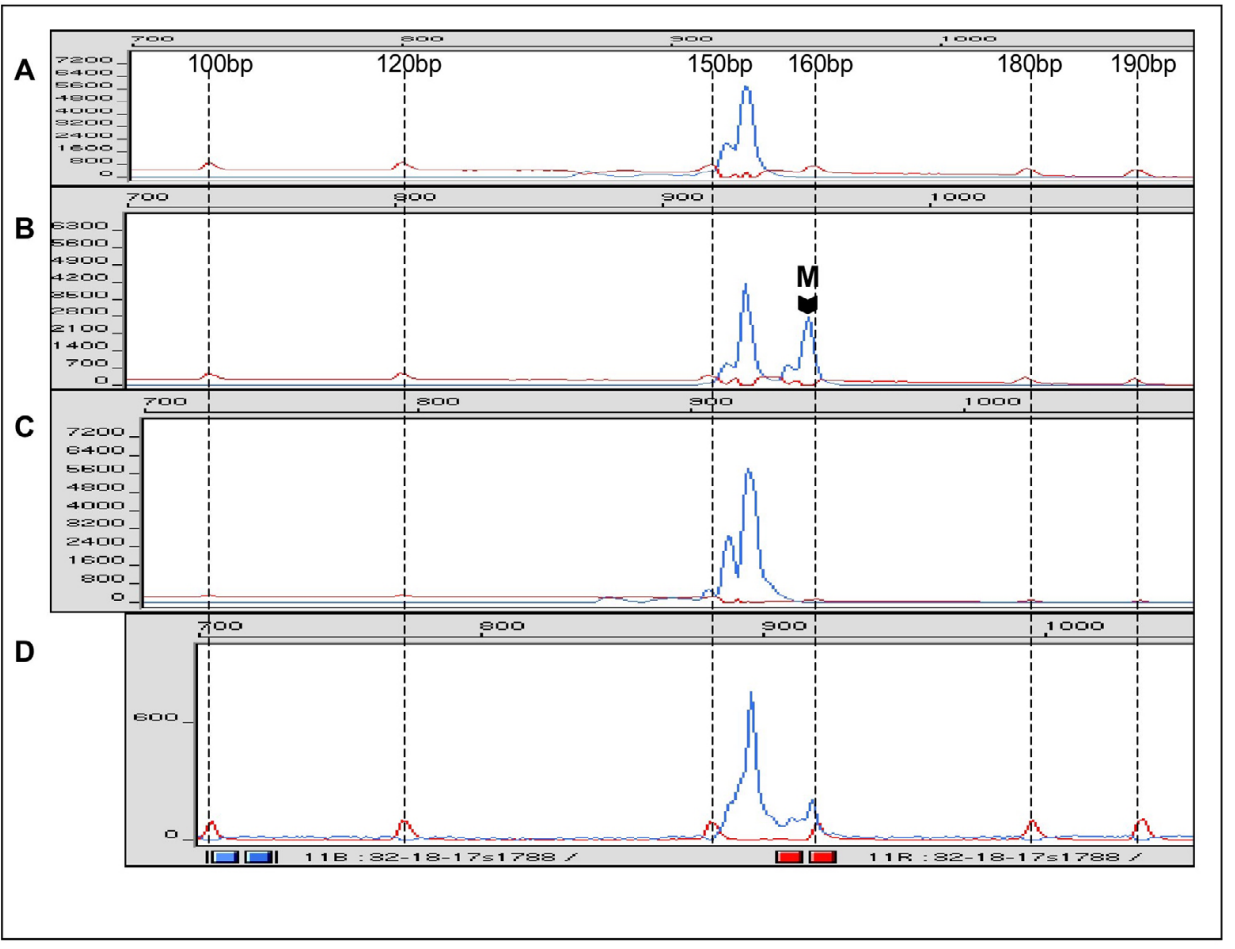
**Electropherogram of PCR with microsatellite D17S1788**. A: PCR with DNA from peripheral blood of the father of the patient (disease carrier). B: PCR with DNA from peripheral blood of the patient. M indicates the maternal allele. C: PCR with DNA from microdissected melanoma cells. D: PCR with DNA from unaffected tissue surrounding the melanoma. Dotted lines indicate peaks and sizes of the molecular size marker GeneScan^®^-400 HD [ROX].

**Figure 3:**
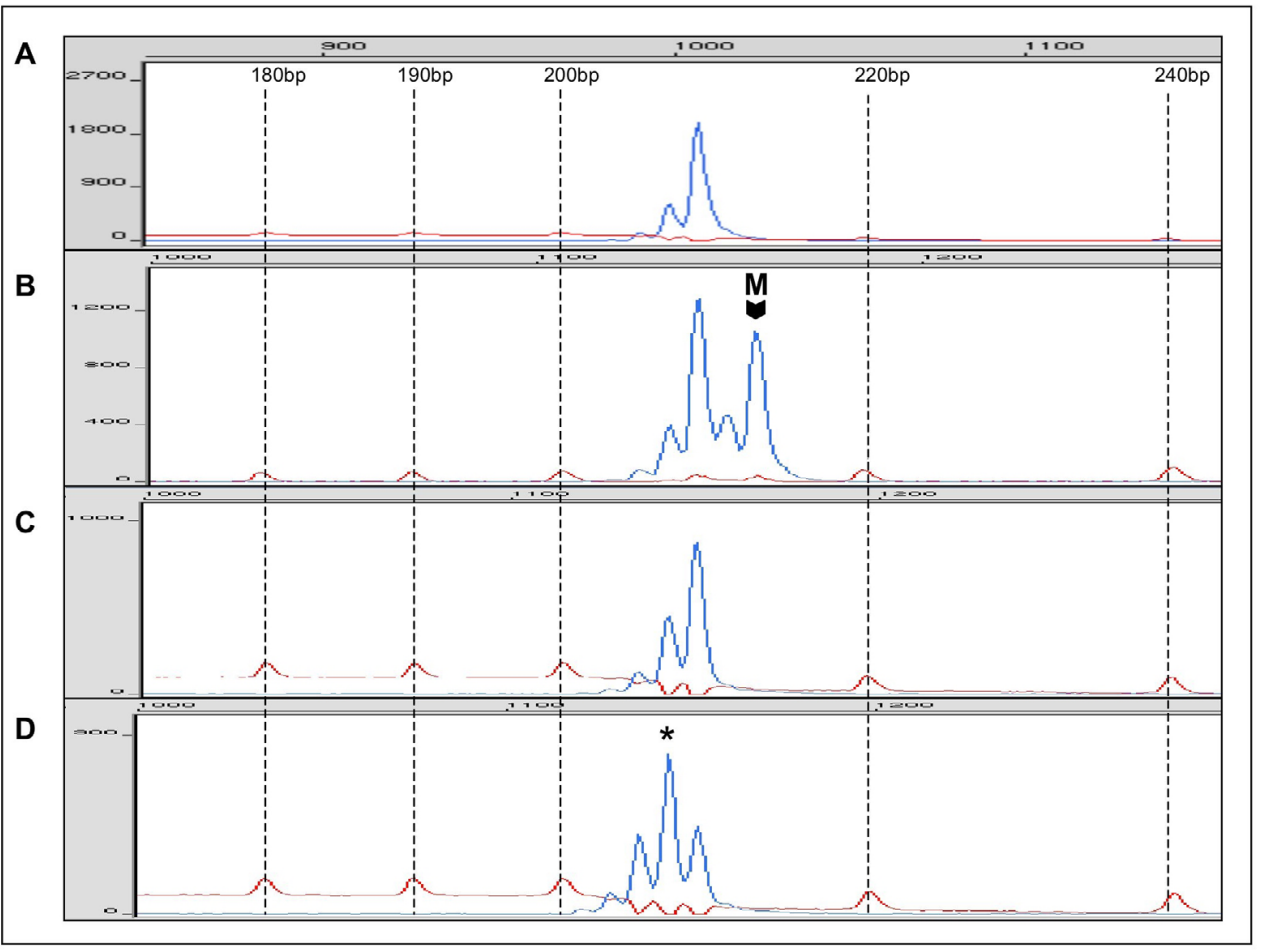
**Electropherogram of PCR with microsatellite IVS27AC28**. A: PCR with DNA from peripheral blood of the father of the patient. B: PCR with DNA from peripheral blood of the patient. M indicates the maternal allele. C,D: PCR with DNA from microdissected melanoma cells showing LOH of the maternal allele (C) and a PCR product of reduced size (D, asterisk). Dotted lines indicate peaks and sizes of the molecular size marker GeneScan^®^-400 HD [ROX].

DNA isolates obtained from blood of the patient and of the patient's farther were analyzed at three microsatellite markers within the *NF1 *gene and four microsatellite markers located near *NF1 *on the long arm of chromosome 17. Only microsatellites D17S1788 and IVS27AC28 were heterozygous in DNA obtained from the patient's blood. Comparison of microsatellite allele sizes in DNA from the patient and from his parents allowed identification of paternal and maternal microsatellite alleles in the patient's DNA (Fig. 2, Fig. 3). For microsatellite D17S1788 located near *NF1*, the paternal allele displayed a size of 154 base pairs (bp) whereas the maternal allele was 159 bp long. The farther was homozygous with an allele of 154 bp and the mother was heterozygous with alleles of 159 bp and 163 bp. For microsatellite IVS27AC28 within intron 27 of the *NF1 *gene, the paternal allele was 208 bp long and the maternal allele was 213 bp long. The farther was homozygous with an allele of 208 bp while the mother displayed alleles of 208 bp and 213 bp.

### LOH analysis at D17S1788 with microdissected melanoma DNA

PCR for microsatellite D17S1788 was performed with DNA obtained from microdissected melanoma tissue. In order to facilitate microscopic identification of melanoma cells, microdissection was performed with a liquid amylopectine-based coverslip (Fig. 1B). PCR amplification was repeated six times. Three PCR amplifications only demonstrated the paternal allele in the melanoma DNA (Fig. 2C). One PCR amplification showed the paternal allele together with a PCR product of smaller size which was considered either microsatellite instability or a PCR artefact (data not shown). The remaining two PCR did not yield a product. In addition, PCR was performed with DNA obtained from tissue surrounding the melanoma on the histological slide in order to rule out that the maternal allele of higher molecular weight could not be efficiently amplified due to formalin-fixation. Figure 2D demonstrates the presence of both alleles although the maternal allele is reduced in intensity as a consequence of DNA degradation induced by formalin fixation. The data suggested loss of the maternal microsatellite allele in the melanoma and probably inactivation of the maternal wild-type *NF1 *gene by deletion but false positive LOH due to stochastic errors induced by PCR could not be excluded.

### LOH analysis by digital PCR at IVS27AC28 with microdissected melanoma DNA

**Figure 4:**
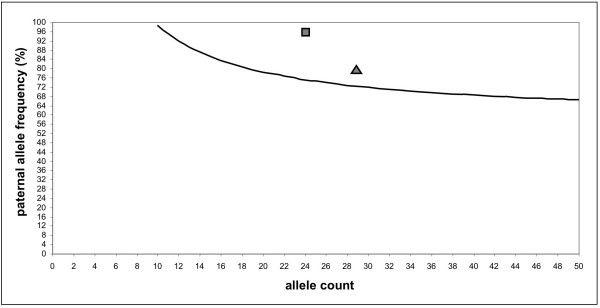
**Graphic representation of SPRT**. The curve shows the boundary for a threshold likelihood ratio of 8 and for a given number of analyzed alleles. Points above this curve are interpreted as detection of statistically significant loss of the maternal allele. The square represents the 23/24 data point while the triangle localizes the 23/29 value.

In order to obtain statistically significant LOH data, digital PCR was performed for microsatellite marker IVS27AC28 located within the *NF1 *gene. Originally, digital PCR was developed for LOH detection by analyzing single nucleotide polymorphisms (SNP) [15] but digital PCR was also used for quantification of microsatellite DNA [23]. The principle of digital PCR consists in analyzing a DNA solution containing a limited number of template molecules by performing multiple PCR amplifications where each PCR contains on average <0.5 template molecules. This approach ensures that most PCR amplifications yielding a specific product started with only one template molecule. Thus, the number of positive PCR amplifications reflects the number of amplifiable template molecules in the examined DNA solution [24]. In the case of the analyzed NF1-associated melanoma, DNA of seven consecutive microdissected melanoma sections representing approx. 3500 tumor cells was pooled and distributed into 184 individual PCR tubes. PCR was performed for microsatellite marker IVS27AC28 and the amplicons of each PCR were analyzed in an automated sequencer in order to determine the size of the microsatellite alleles. 28 PCR amplifications (15%) demonstrated microsatellite DNA and of these positive reactions 22 (79%) contained only the paternal allele of 208 bp size (Fig. 3C). One PCR yielded both, the paternal and maternal alleles and five reactions (7%) demonstrated alleles of different sizes, most probably representing PCR artefacts as allele peaks were found within the range of microsatellite stutter bands of the wild type alleles (Fig. 3D). Based on all detected alleles (n = 29), digital PCR detected 23 paternal alleles (79%) while the maternal alleles or aberrant alleles were found in 6 reactions. Excluding the aberrant PCR products, the paternal allele was present in 96% (23/24) of all reactions. In both cases, the statistical analysis with the sequential probability ratio test (SPRT) revealed that LOH with loss of the maternal allele in the analyzed melanoma had a likelihood ratio greater than 8, thus confirming the observation. The boundary values for the allele frequencies which had to be exceeded for statistical significance were 72% for 29 detected alleles and 75% for 24 alleles. Figure 4 gives a graphic representation of the SPRT analysis. In the formalin-fixed tissue surrounding the melanoma, digital PCR of microsatellite marker IVS27AC28 detected 11 paternal and 13 maternal alleles demonstrating that no allele loss was present in the skin adjacent to the melanoma.

## Discussion

The study presents the first molecular evidence of inactivation of both copies of the *NF1 *gene in a typical superficial spreading melanoma of a patient with neurofibromatosis type 1. The paternal allele bears the germline mutation *NF1 *c. 5556 G/A as the father of the patient is the disease carrier. The maternal allele is deleted in the tumor tissue as demonstrated by loss of heterozygosity of a microsatellite marker located within the *NF1 *gene. No LOH of the analyzed microsatellite markers could be detected in the tissue surrounding the melanoma. The inactivation pattern of the *NF1 *gene in the analyzed melanoma represents the classical double-hit inactivation of a tumor suppressor gene as postulated by Knudson in 1971 [25]. Deletion of the *NF1 *gene in NF1-associated melanoma suggests that the NF1 genetic background promoted melanoma genesis. This assumption is strengthened by the observation that the melanoma occurred at the unusually young age of 15 years and by the detection of the gene deletion in an early disease stage melanoma. It has been reported previously that melanoma in NF1 tends to arise at younger age [11] and mutation detection in an early tumor stage is a stronger indication of a causative role of the detected mutation than mutation detection in a late stage malignant tumor.

Despite the molecular findings supporting a melanoma promoting genetic background in the analyzed NF1 case, the question remains why melanoma incidence is not markedly elevated in NF1. This apparent inconsistency may be explained by the melanocyte abnormalities found in NF1 patients, by melanoma histogenesis and by divergent tumor evolution pathways in malignant melanoma.

Increased macular pigmentations visible in café-au-lait-macules (CALMs) and in axillary freckling are among the first clinical signs of NF1 [10]. It has been hypothesised that formation of CALMs might be caused primarily by the effect of neurofibromin haploinsufficiency on melanocyte differentiation and pigmentation and less by an effect on cell proliferation. It could be demonstrated that neurofibromin colocalizes with melanosomes [26] and melanocyte density seems only moderately and inconsistently elevated in NF1-associated CALMs [10,27]. Furthermore, cell studies with melanocytes of NF1 patients have not detected enhanced RAS-GTP-levels, which would have been indicative of an enhanced melanocyte proliferation [28].

On the other hand, the round shape of CALMs is compatible with a monoclonal melanocyte expansion within the skin and analysis of X-inactivation pattern indeed suggests a monoclonal origin [29]. Nevertheless, the monoclonal origin of CALMs may be explained sufficiently by melanocyte differentiation during embryogenesis and does not require enhanced melanocyte proliferation. Most knowledge on melanocyte differentiation during embryogenesis has been obtained from the murine system. Melanoblasts, which are the melanocytes precursors, migrate in mice from the neural crest dorsolaterally and enter the skin where they proliferate clonally and finally differentiate into mature skin melanocytes [30,31]. One may assume that modulation of pigmentation by neurofibromin haploinsufficiency during regular melanoblast expansion in the skin leads to formation of monoclonal and round shaped CALMs.

The lack of *NF1 *allele loss in CALMs [29] and the observation that melanomas arising in NF1 patients do not demonstrate a preferential association with CALMs [11] further support the view that neurofibromin does not seem to control proliferation of mature skin melanocytes. Knowledge is still incomplete on homeostasis and regeneration of skin melanocytes but in the murine system, hair follicle melanocytes are provided by melanocyte stem cells located in the lower permanent portion of the hair follicle [32] and a similar system of melanocyte stem cells may be responsible for melanocyte regeneration in humans as well. As CALMs patterns appear stable after the first decade it is not probable that neurofibromin haploinsufficiency may strongly affect skin melanocyte regeneration.

Assuming that neurofibromin does not control proliferation of mature skin melanocytes or putative melanocyte stem cells, one may not expect that the NF1 genetic background enhances melanoma development from these cells.

A significant portion of melanomas do not arise in normally pigmented skin but develop from melanocytic nevi which are present at birth or are acquired later in life [33]. The true identity of the precursor cells of melanocytic nevi remains elusive but it is assumed that congenital melanocytic nevi (CMN) develop during embryogenesis from neural crest derivatives which retain a certain ability to segregate into different neural crest lineages as CMN may contain cells displaying neurogenic differentiation patterns [34,35]. Likewise, acquired melanocytic nevi are thought to origin from melanocyte precursors which show wider differentiation plasticity than mature skin melanocytes [36,37]. It can be speculated that melanocytic nevi displaying a neurogenic differentiation type may retain neurofibromin control of cell proliferation and may, therefore, be susceptible to malignant transformation in a NF1 genetic background. Nine of the 37 NF1-associated melanoma cases reported in the literature were associated with giant congenital melanocytic nevi [11] which supports the hypothesis that nevi might be the primary source of NF1-associated melanomas. Likewise, the melanoma of our patient most probably developed from an acquired melanocytic nevus. The importance of a neurogenic differentiation pattern for NF1-associated melanoma genesis is further demonstrated by the detection of frequent *NF1 *allele loss in desmoplastic neurotropic melanoma [12] which may display neural features and markers [38,39].

The existence of distinct and divergent tumor evolution pathways in malignant melanoma [40] may serve as a complementary explanation for the apparent lack of a grossly elevated melanoma incidence in NF1. It could be demonstrated that more than 80% of all melanomas contain mutations in BRAF or N-RAS [41]. Moreover, BRAF mutations could be detected in 82% of all melanocytic nevi [42]. As neurofibromin functions as a negative regulator of the RAS-RAF-MEK-ERK-pathway [3], selective pressure for *NF1 *loss should be absent in premalignant melanocytic proliferations harbouring activating mutations of BRAF or N-RAS which represent the majority of all acquired melanocytic nevi.

Taken together, neurofibromin function in mature skin melanocytes, melanoma histogenesis as well as genetic tumor evolution pathways suggest that neurofibromin loss or haploinsufficiency may enhance melanoma risk only in minor subset of all melanocytic cells which may progress to melanoma. Nevertheless, congenital or acquired melanocytic nevi could share an enhanced melanoma risk in NF1 patients.

The presented data were generated by PCR analysis of microdissected tissue obtained from a thin superficial spreading malignant melanoma which was formalin-fixed and paraffin-embedded. Molecular analysis of this material faces specific problems. It has been observed, that formalin fixation leads to a bias when microsatellite alleles are detected and quantified by conventional PCR amplification [17]. When two alleles differing in size are present in formalin-fixed tissue, microsatellite PCR tends to amplify preferentially the allele of smaller molecular size. This may lead to false positive detection of loss of heterozygosity (LOH). A second technical limitation is related to the low number of PCR-amplifiable DNA sequences within DNA obtained from formalin-fixed tissue. It could be demonstrated that the fraction of PCR-amplifiable DNA can be as low as ≈1 to 3600 [18]. Especially when using microdissected formalin-fixed tissue, only few target molecules may be present for microsatellite PCR and allele ratios may not be determined correctly due to stochastic error. Both problems can be circumvented by using digital PCR for quantification of microsatellite allele ratios. Digital PCR seeks to amplify allele sequences starting from one target molecule. In case of heterozygous microsatellite DNA, each individual and successful PCR amplification should begin with one molecule of the allele of lower molecular size or with one molecule of the allele of higher molecular size. As allele ratios are calculated based on the number of positive PCR amplifications for each allele, differences between allele amplification efficiencies do not bias allele quantification. Moreover, digital PCR reports the number of amplifiable alleles contained in the analyzed DNA solution which allows correct statistical interpretation of the results. In the case of the analyzed NF1-associated melanoma, 29 alleles could be detected in DNA obtained by microdissection of approx. 3500 cells. The paternal allele was present in 79% of all PCR amplifications and LOH detection was statistically significant based on the sequential probability ratio test.

## Conclusion

We could demonstrate the inactivation of both copies of the *NF1 *gene in a typical superficial spreading melanoma of a patient with neurofibromatosis type 1 which supports a non-fortuitous association of melanoma and NF1. Therefore, melanoma screening of NF1 patients may be recommendable even if the NF1 genetic background may not result in a strongly enhanced melanoma incidence. Nonetheless, congenital or acquired melanocytic nevi in NF1 patients may carry an enhanced melanoma risk. In our study, analysis of *NF1 *allele status in a formalin-fixed melanoma specimen necessitated the combination of liquid coverslip laser microdissection, microsatellite analysis and digital PCR. This novel technical approach may be indispensable when examining allele ratios in rare formalin-fixed clinical specimens as it allows quantitative and statistically controlled detection of low abundance DNA or RNA target sequences.

## Methods

### Laser microdissection with liquid coverslip

Laser microdissection with liquid coverslip was performed as previously described [14]. Briefly, formalin-fixed tissue was cut into 10 μm thick sections and mounted on superfrost slides. The sections were deparaffinized with xylole and were then H&E-stained. The slides were covered with a liquid coverslip made of 0.04 % amylopectine and 10% bovine albumin (Sigma-Aldrich, Munich, Germany). After vacuum-drying, a total of 10 consecutive sections were microdissected at multiple areas (approx. 50000 μm^2^) in order to obtain approx. 500 melanoma cells per section (Fig. 1B). Laser microdissection was carried out using the automated P.A.L.M. Robot, MicroBeam system (P.A.L.M., Bernried, Germany). Microdissected tissue was catapulted onto thin 5 × 5 mm polyester films (Gelbond PAG, BioWhittaker Molecular Applications, Rockland, ME, USA).

### DNA extraction

Polyester films carrying the microdissected tissue were placed into Eppendorf caps. Eppendorf caps contained 20 μl of ATL tissue lysis buffer with 2.5 mg/ml proteinase K (QIAamp DNA Micro Kit, Qiagen, Hilden, Germany). Tissue digestion was carried out overnight at 37°C in 0.2 ml Eppendorf tubes. DNA extraction from this solution was performed with the QIAamp DNA Micro Kit according to the manufacturer's instructions except for DNA elution from spin columns which was performed in a volume of 25 μl of H_2_O. Whole blood was obtained from the boy and from the boy's farther for DNA extraction with the QIAamp DNA Mini Kit.

### Microsatellite loss of heterozygosity (LOH) analysis

The following microsatellite loci were analysed: 53.0A/53.0B, NF1.PCR3, IVS27AC28, which are located within the *NF1 *gene, and D17S1699, D17S1788, D17S2204, D17S1824, which are located near *NF1 *on the long arm of chromosome 17. Primer sequences of these microsatellite loci were obtained from The Genome Database [43]. Oligonucleotides were labelled 5' with fluorescent dye (6-FAM) (Sigma-Genosys, Steinheim, Germany). PCR was performed with 2 U/100 μl Taq-polymerase AmpliTaq Gold (Applied Biosystems, Foster City, CA, USA) according to the manufacturer's recommendations. Standard PCR was done in a total volume of 25 μl with 0.5 μl DNA solution obtained from whole blood or with 5 μl DNA solution obtained from microdissected tissue. Amplification was started after Taq-polymerase activation at 95°C for 8 min. The following thermal cycle program was used for all primers: 5 cycles of 1 min. 30 sec. at 55°C, 1 min. at 72°C, 45 sec. at 94,5°C followed by 5 cycles of 1 min. 30 sec. at 52°C, 20 sec. at 72°C, 20 sec. at 94°C followed by 20 cycles (blood) or 35 cycles (microdissected tissue) of 25 sec. at 50°C, 20 sec. at 72°C, 20 sec. at 94°C with a last elongation step of 5 min at 72°C. For digital PCR of melanoma tissue with primers IVS27AC28, 70 μl of DNA solution obtained from 7 microdissected tissue sections were distributed into 184 PCR reactions of 12.5 μl volume. Digital PCR was also performed with DNA obtained from the surrounding non-melanoma tissue of two tissue slides. This extracted DNA was distributed into 48 individual PCR reactions. The following thermal cycle program was used: 10 cycles of 2 min. at 55°C, 1 min. at 72°C, 45 sec. at 94.5°C followed by 5 cycles of 1 min. 30 sec. at 52°C, 20 sec. at 72°C, 20 sec. at 94°C followed by 30 cycles of 25 sec. at 52°C, 20 sec. at 72°C, 20 sec. at 94°C with a last elongation step of 5 min at 72°C. Separation, detection and sizing of PCR products were performed in a 6% acrylamide gel (Gel-Mix6, Gibco BRL, Gaithersburg, USA) using an automated sequencer (Applied Biosystems ABI-PRISM 373A XL) and the molecular size marker GeneScan^®^-400 [ROX]™ (Applied Biosystems).

### Statistical analysis

The result of digital microsatellite PCR was analysed by the sequential probability ratio test (SPRT) as previously described for digital PCR of SNP [15]. The test compares two probabilistic hypotheses as data accumulate [44]. One hypothesis is that the maternal and paternal microsatellite alleles near or within the *NF1 *gene have the same proportion in the melanoma. The second hypothesis states that the maternal microsatellite allele is deleted in the patient's melanoma (LOH). In the first case, the frequency of the paternal allele in the sample is 50%, whereas under the second hypothesis the frequency should be 100%. As it is virtually impossible to isolate pure tumor cells even by microdissection, the assumption is made, that at least 50% of the extracted DNA corresponds to neoplastic tissue which leads to a ratio of ≥ 66.7% for the second hypothesis. The data analysis is shown in figure 4. The curve represents the SPRT analysis for a threshold likelihood ratio of 8 and for a given number of analyzed alleles. Points above this curve are interpreted as loss of the maternal allele where the likelihood ratio of 8 corresponds approx. to a two-sided 95% confidence interval.

## Competing interests

The author(s) declare that they have no competing interests.

## Authors' contributions

AR conceived the study, carried out laser microdissection and digital PCR analysis and drafted the manuscript. BB determined the *NF1 *mutation in the patient and in the farther of the patient. AN provided specimen and histopathologic diagnosis and revised the manuscript critically. All authors read and approved the final manuscript.
